# Protocol for the quantitative characterization of cell aggregates using an MRI setup maintaining optimal cultivation conditions

**DOI:** 10.1016/j.xpro.2025.104002

**Published:** 2025-07-30

**Authors:** Rebecca Wißmann, Petros Martirosian, Marina Danalache, Stefanie Elser, Fritz Schick

**Affiliations:** 1 Department of Diagnostic and Interventional Radiology, University Hospital of Tübingen, 72076 Tübingen, Germany; 2 Section on Experimental Radiology, Department of Diagnostic and Interventional Radiology, Tübingen University Hospital, Tübingen, Germany; 3 Department of Orthopaedic Surgery, University Hospital of Tübingen, 72076 Tübingen, Germany

**Keywords:** Cell culture, Cancer, Organoids, Tissue Engineering, Physics

## Abstract

The use of destructive biochemical assays and the preparation of histologic samples are routinely employed to monitor development and viability of 3D cell aggregates. Magnetic resonance imaging (MRI) offers a non-destructive, high-resolution alternative to histological analysis, enabling longitudinal assessment of cellular dynamics while preserving sample integrity. Here, we present a protocol for non-invasive MR imaging of cell spheroid clusters by creating an adequate imaging environment. We describe steps for spheroid formation, casting of the imaging tube, cell cultivation, and data evaluation.

For complete details on the use and execution of this protocol, please refer to Wißmann et al.^1^

## Highlights

• Steps for creating spheroid samples for visualization via 3T MRI scanner
• Step-by-step instructions for creating the MR-compatible experimental setup
• Detailed description of the MR protocols
• Quantification of relaxation times, parameter mapping, and calculation of ADC and MTR

## Abstract (editor highlights)

Publisher’s note: Undertaking any experimental protocol requires adherence to local institutional guidelines for laboratory safety and ethics.

## Graphical abstract

**Figure:**
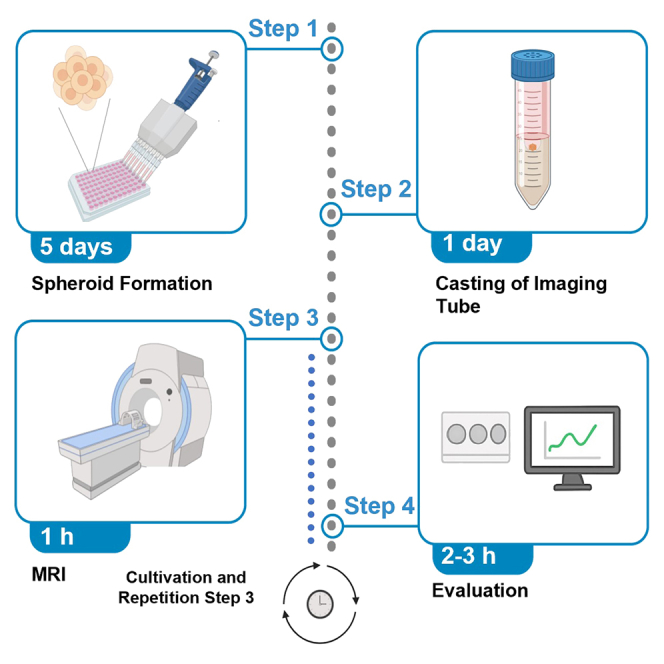

## Before you begin

Three-dimensional cell cultures, particularly those in the form of spheroids, have been shown to offer a more physiologically relevant model than traditional 2D cultures,^2^^,^^3^^,^^4^ as they are able to mimic the complex interactions that are observed *in vivo*, including cell-cell and cell-matrix interactions.^5^^,^^6^^,^^7^ Spheroids exhibit a unique structure with distinct zones of proliferation, quiescence, and necrosis.^8^^,^^9^ This heterogeneity, along with their gene expression profiles, closely resembles that of avascular tumors, making them valuable tools for preclinical research, drug screening, and regenerative medicine applications.^10^^,^^11^^,^^12^

While microscopy techniques provide valuable insights, they often require invasive procedures like sectioning.^13^^,^^14^ This is also the case for destructive biochemical assays. MRI offers a non-invasive alternative by analyzing parameters like T1, T2, the apparent diffusion coefficient (ADC), and magnetization transfer ratio (MTR) to characterize spheroid properties, including cell viability and tissue composition, without disrupting their structure.^1^

This protocol demonstrates the application of various MRI techniques for non-invasive characterization of cell spheroids and other cell aggregates. In comparison with the time-consuming preparation of histological samples for microscopy, this method significantly reduces preparation time. By facilitating serial MRI acquisitions under optimized cultivation conditions, the technique mitigates structural perturbations associated with repeated handling, thereby maintaining the native spatial architecture of the spheroids throughout the experimental timeline.

### Preparations and considerations

1. Familiarize yourself with the needs and requirements of your cell line, this protocol is exemplary for SW1353 chondrosarcoma cells.
2. All cell culture should be performed under sterile conditions and equipment following local guidelines for biosafety and good laboratory practice.
3. Make sure that the MRI scanner used is also approved for the corresponding safety level of the cell lines.

### Thawing cells from cryogenic storage and cell culture

**Timing: 3–5 days**

This step describes how cells are thawed from cryogenic storage and kept in culture.

4. Thawing cells from cryogenic storage.
  a. Preheat water bath to 37°C.
    i. Warm up a sufficient amount of cell culture thawing media (for details please refer to materials and equipment setup): 10 mL per cryo vial + 8-15 mL for a T75 flask.
  b. Transfer 10 mL of warm cell culture media into a 50 mL conical tube.
  c. Remove cells from cryogenic storage and place vial into water bath.
  d. Once the cells are almost fully defrosted, transfer the contents of the vial into the prepared 50 mL conical tube.
  e. Centrifuge the conical tube to pellet cells for 3 min at 300 x g.
  f. Discard the supernatant and resuspend the pellet in 8–15 mL of thawing medium.
  g. Transfer the resuspended cells into a T75 culture flask.
  h. Place the flask into an incubator (37°C, 5% CO_2_, normoxia).

**CRITICAL:** Due to high DMSO concentrations in cryo-medium, handling should be swift once defrosting commences, transportation on ice is recommended.

***Note:*** After the cells have been cultivated to confluence in a T75 flask, it is recommended to switch to a T175 flask to increase yield.

5. Cell Passaging.
  a. Ensure 80%–90% confluency under a microscope.
  b. Pre-heat in a water bath to 37°C:
    i. Cell culture media (for details please refer to materials and equipment setup).
    ii. PBS w/o calcium and magnesium.
    iii. Trypsin.
  c. Remove previous media.
  d. Wash cells carefully with 10 mL PBS (T175).
  e. Add 7 mL of trypsin and place it into an incubator at 37°C for 5 min.
  f. Check cell detachment under a microscope.
  g. Add 7 mL of culture media and carefully transfer the cell suspension into a 50 mL conical tube.
  h. Centrifuge the conical tube to pellet cells for 3 min at 300 x g.
  i. Resuspend the pellet with 3 mL culture media.
  j. Prepare a T175 flask with 30 – 50 mL cell culture media.
  k. Transfer 1 mL of the cell suspension into the T175 flask.
  l. Place the flask into an incubator (37°C, 5% CO_2_, normoxia).

### Spheroid generation

**Timing: 5 days**

This step outlines the process for forming spheroids.

***Note:*** This protocol is for a spheroid size of approximately 65,500 cells. In total 12 x 10^6^ cells (= 192 spheroids) will be needed for MR imaging. Make sure you have enough cells before commencing, three T175 flasks should suffice.

6. Preparation of Cell Culture.
  a. Pre-heat in a water bath to 37°C:
    i. Cell culture media.
    ii. PBS w/o calcium and magnesium.
    iii. Trypsin.
  b. Remove previous media.
  c. Wash cells carefully with 10 mL PBS.
  d. Add 7 mL of trypsin and place it into an incubator at 37°C for 5 min.
  e. Check cell detachment under a microscope.
  f. Add 7 mL of culture media and carefully transfer the cell suspension into a 50 mL conical tube.
  g. Centrifuge the conical tube to pellet cells for 3 min at 300 x g.
  h. Resuspend the pellet with 10 mL culture media.
  i. Repeat the steps above for all cell culture flasks.
  j. Mix all cell suspensions.
  k. Check cell count of suspension (e.g. with an automatic counter or with a hemocytometer).
  l. Adjust the final cell count of the suspension to 3.125 x 10^5^ cells/mL by diluting with cell culture medium, a total of 40 mL will be needed.

***Note:*** If the cell count is too low for dilution, pellet the cells again by centrifugation and resuspend in a lower amount of culture media.

7. Seeding the cells.
  a. Prepare 2 x 96-well ultra-low attachment plates.
  b. Mix cell suspension by inverting the conical tube.
  c. Add 200 μL of cell suspension per well.
  d. To facilitate aggregation, centrifuge the plates for 5 min at 300 x g.
  e. Incubate plate for 5 days under cell culture conditions.

**CRITICAL:** As cells sink to the bottom of the tube, it is essential to mix the suspension regularly to ensure an even spheroid size throughout the wells.

***Note:*** The ability to form cell spheroids varies significantly among different cell types, as spheroid formation is highly dependent on intrinsic cellular adhesion properties, extracellular matrix interactions, and specific culture conditions. Consequently, not all cell populations exhibit equal propensity for self-assembly into three-dimensional aggregates, necessitating cell type-specific optimization of culture parameters to ensure reproducible spheroid formation. For more details, refer to troubleshooting 1.

## Key resources table

**Table:** 

REAGENT or RESOURCE	SOURCE	IDENTIFIER
**Chemicals, peptides, and recombinant proteins**		

Dulbecco’s phosphate-buffered saline	Gibco, Thermo Scientific, Waltham, MA, USA	Cat# 14040091
Agarose	Thermo Scientific, Waltham, MA, USA	Cat# 17850
Dulbecco’s modified Eagle’s medium/nutrient mix F-12	Gibco/Thermo Fisher Scientific, Waltham, MA, USA	Cat# 11330032
Fetal bovine serum	Gibco/Thermo Fisher Scientific, Waltham, MA, USA	Cat# A5256701
Penicillin streptomycin (10,000 U/mL)	Gibco, Thermo Scientific, Waltham, MA, USA	Cat# 15140122
Trypsin	Lonza Group AG, Basel, Switzerland	Cat# CC-5012

**Experimental models: Cell lines**		

SW1353	ATCC	Cat# HTB-94

**Software and algorithms**		

CASY Model TT Cell Counter and Analyzer	Roche Diagnostics GmbH, Mannheim, Germany	N/A
MAGNETOM Prisma Fit	Siemens Healthineers, Erlangen, Germany	N/A
Software Application “MapIt”	Siemens Healthineers, Erlangen, Germany	N/A
Image J Version 1.51	National Institutes of Health, USA	RRID: SCR_003070 https://imagej.net/ij/
GraphPad Prism version 10.1.1	GraphPad Software Inc., San Diego, CA, USA	RRID: SCR_002798 https://www.graphpad.com/

**Other**		

Thermo Nunclon Sphera plates	Thermo Scientific, Waltham, MA, USA	Cat# 174925
50 mL tube	Greiner Bio-One International GmbH, Kremsmünster, Austria	Cat# 210270
CELLSTAR cell reactor tube	Greiner Bio-One International GmbH, Kremsmünster, Austria	Cat# 227245
14 mL tube	Greiner Bio-One International GmbH, Kremsmünster, Austria	Cat# GB191161
75 cm^2^ cell culture flask	Greiner Bio-One International GmbH, Kremsmünster, Austria	Cat# 658940
175 cm^2^ cell culture flask	Greiner Bio-One International GmbH, Kremsmünster, Austria	Cat# 660160
EASYstrainer 100 μM for 50 mL tubes	Greiner Bio-One International GmbH, Kremsmünster, Austria	Cat# 542000
Plastic box (1 L, 12 × 6 × 10 cm)	N/A	N/A

## Materials and equipment

**Table:** Cell culture medium

Reagent	Final concentration	Amount
Advanced Dulbecco’s modified Eagle medium with F12 (DMEM/F12)	89%	445 mL
Fetal Bovine Serum	10%	50 mL
Penicillin/ Streptomycin	1%	5 mL
**Total**	**–**	**500 mL**

Cell culture medium is to be stored at 4°C and used within six weeks.

**Table:** Thawing medium

Reagent	Final concentration	Amount
Advanced Dulbecco’s modified Eagle medium with F12 (DMEM/F12)	79%	39,5 mL
Fetal Bovine Serum	20%	10 mL
Penicillin/Streptomycin	1%	500 μL
**Total**	**–**	**50 mL**

Thawing medium is to be stored at 4°C and used within six weeks.

**Table:** 1% (w/v) agar for cultivation tube

Reagent	Final concentration	Amount
Dulbecco’s Phosphate Buffered Saline	99%	500 mL
Agar	1%	5 g
**Total**	–	**500 mL**

Cultivation tubes should be stored at 4°C and can be used for approximately four weeks.

**Table:** MRI protocols

	PD/T2-w	T1-w	T1-map	T2-map	DWI	MTR
Sequence name^a^	2D TSE	3D VIBE	3D VFA VIBE	SE_MC	RESOLVE	3D GRE
Echo train length	18	1	1	32	1	1
TR (ms)	3000	6.8	9.2	3000	3000	25
TE (ms)	12/160	2.74	2.71	10–320	47	3.18
Flip angle (deg)	90-180_n_	10	2/8/15	90-180_n_	180	10
BW (Hz/Px)	191	190	190	235	744	190
Matrix	120×160×15	173×256x128	120×160×30	120×160×1	120×160×1	120×160×30
FOV (mm^3^)	120×160×16	116×154×38	120×1600×15	120×160×2	120×160×3	120×160×15
Voxel size (mm^3^)	1.0×1.0×1.0	0.6×0.6×0.6	1.0×1.0×1.0	1.0×1.0×2.0	0.5×0.5×3.0	0.5×0.5×0.5
Scan time (min:s)	3:41	4:28	5:37	6:05	6:53	4:48×2

a The absence of standard nomenclature for each common type of sequence is attributable to the use of unique terminology by manufacturers to name their sequences. In this study, Siemens Healthineers Products have been employed. The following table provides a list of the equivalent manufacturer’s acronyms and the corresponding type of sequence.^15^

## Step-by-step method details

In the following sections, we describe the preparation of the imaging tube which will create a suitable environment for cell spheroids, followed by setup instructions for the scanner. Furthermore, we give details instructions on the possible readouts.

### Preparation of the imaging tube

**Timing: 1 day**

This step describes the casting and assembly of the imaging tube.

***Note:*** For imaging a cylindrical 50 mL conical tube is used in order to ensure a homogeneous magnetic field distribution, thereby minimizing susceptibility to artefacts and field inhomogeneities that could compromise image quality. To maintain optimal imaging conditions, spheroid clusters must be immobilized and surrounded (up to a distance of approximately 1–2 cm) by a material with homogeneous MR signal intensity and suitable magnetic properties (as agar and culture media). Moreover, the entirety of the sample must be accommodated within a relatively compact and uniformly distributed radiofrequency (RF) coil for signal recording purposes. This tube configuration simultaneously supports long-term cell cultivation, allowing for repeated imaging over extended periods while preventing structural disruption of the spheroids due to repeated pipetting or handling.

**Figure 1:**
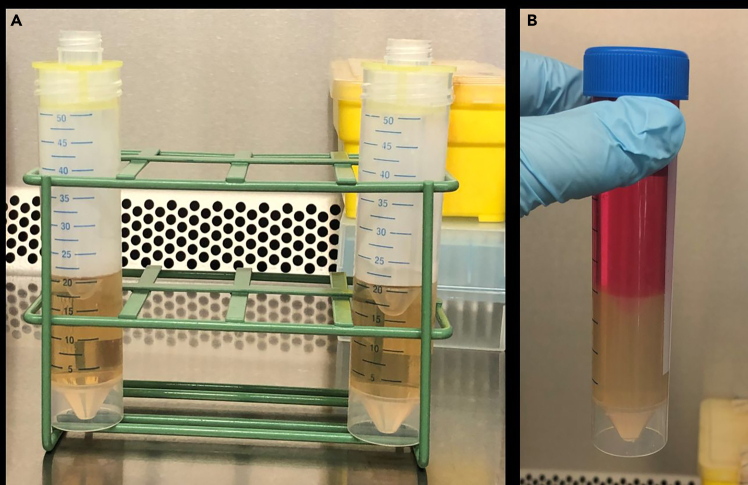
Preparation of the imaging tube (A) Casting of the indentation with a 14 mL round bottom culture tube. (B) Final imaging tube with 1% agarose gel and cell culture medium.
5. Let the agar gel solidify.
6. Remove the small tube carefully.
7. Carefully transfer 192 single spheroids into the indentation of the agar.
8. Fill the conical tube completely with pre-warmed cell culture medium (approximately 35 mL).

***Note:*** Has the agarose gel already solidified after autoclaving, it can be reheated in a microwave.

1. Preparation of the 1% agarose gel:
  a. Mix 5 g agarose in 500 mL sterile PBS in an autoclavable 500 mL screw top glass bottle.
  b. Autoclave the solution according to the liquids protocol of the available autoclave.
2. Carefully transfer 20 mL of liquid agarose into a 50 mL conical tube, avoid the formation of air bubbles.
3. Create an approx. 2 cm deep indent with a smaller 14 mL round bottom culture tube.
4. Fixate the round bottom tube either with a sterile strainer or parafilm (Figure 1).

**CRITICAL:** Take care when handling hot agarose gel. Ensure the bottle is loosely capped when heating to avoid pressure build-up.

***Note:*** Ensure full immersion of the sample to maintain optimal physiological conditions and prevent air bubble formation, which could interfere with imaging quality. For imaging use the standard lid, for cell cultivation exchange lid with a ventilated top.

### MR imaging of cell spheroid clusters

**Timing: 1 h**

This step describes the positioning of the tube image acquisition on an 3 Tesla MRI scanner.

***Note:*** Modern quantitative MRI techniques, such as pixel-by-pixel mapping of T1 and T2 relaxation times, ADC, and MTR, provide valuable insights into tissue composition and characteristics. Relaxation times, T1 and T2, are influenced by the composition of the intra- and extracellular compartments, including the presence of ions and molecules with varying sizes, structures, and mobilities, and their interactions with water molecules, which are the primary source of the MR signal.^16^ Diffusion-weighted imaging (DWI), which assesses ADC, is sensitive to microscopic obstacles, such as cell organelles or membranes that hinder the free movement of water molecules. This sensitivity allows DWI to detect changes in cell size, integrity, and viability.^17^ Magnetization Transfer Ratio (MTR) reflects the interaction between mobile water molecules and macromolecules. This interaction can occur through various mechanisms, such as proton exchange or the exchange between bound water within macromolecular shells and free water molecules.^18^

***Note:*** Make sure that the image tube has cooled down to a temperature of 21 degrees. The transfer time from lab to scanner may be sufficient.

**Figure 2:**
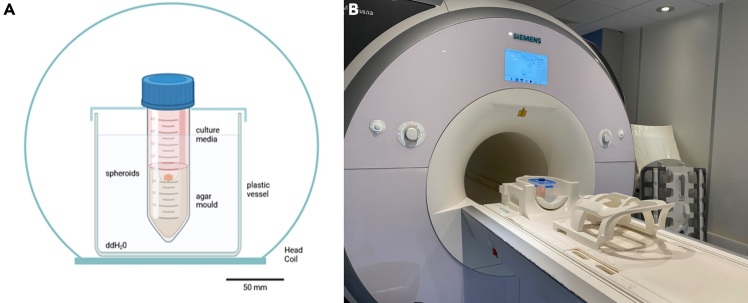
Experimental setup (A) Visualization of SW1353 cell spheroids in a mold of 1% agar and culture media, placed in a plastic box, surrounded by ddH_2_O and placed into the head coil of a 3 Tesla MRI scanner.^1^ (B) Placement within the scanner (MAGNETOM Prisma Fit).
11. Move the table into the scanner.
12. Start your imaging /MR protocols.
13. Save your acquired data in DICOM-format.
14. Remove the cultivation tube.
15. Change the standard lid with the ventilated cap of the cell reactor tube.
16. Store spheroids in an incubator (37°C, 5% CO_2_, normoxia) if further use or repeated examination is desired.

***Note:*** Double distilled H_2_O is used to ensure that there is absolutely no residue (e.g. rust) which might cause artefacts in imaging.

***Note:*** The box should stand upright and be fixed in such a way that no movement is possible during the measurement.

9. Prepare imaging box.
  a. Fill a plastic container with 21°C warm double distilled water (ddH_2_O).
  b. Add the imaging tube.
  c. Parafilm can be used to fix the tube in place, another option is to drill a ø 30 mm hole in the lid of the plastic container.
10. Place the box into the head coil (Figure 2) and close it.

**CRITICAL:** The temperature has a strong influence on MR features and should therefore be closely monitored and remain constant during subsequent measurements. Troubleshooting 2.

### Image processing

**Timing: 2 h**

This step describes the evaluation of the previously collected image data.

***Note:*** The subsequent processing of the acquired image data facilitates the reading process. The usual MR images with different gray values show the signal intensity of the examined sample. Often, details can be depicted somewhat better with pseudo color images, as described below. Quantitative assessment of features of the specimen can be achieve by calculation of color-encoded parameter maps from sets of recorded images with appropriate parameters (T1 and T2 relaxations times, ADC, or MTR). Furthermore, this process enables the extraction of quantitative data, which is shown here using the open-source freeware Image J. This data can then be analyzed using statistical methods.

17. Obtaining Relaxation Times.
  a. Start Image J.
  b. Load (e.g. T1-) images and select slices with spheroids.
  c. Select the spheroid cluster with the oval or freehand selection.
  d. Open Analyze > Tool > ROI Manager (Figure 3).

**Figure 3:**
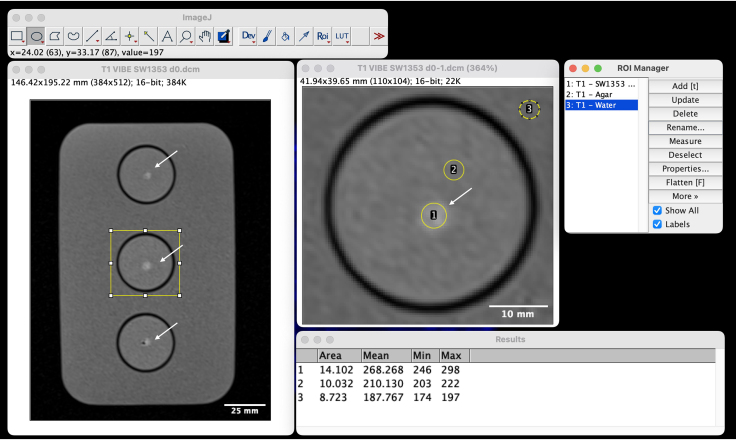
Selecting regions of interest (ROIs) of a 3D VIBE image in Image J to obtain T1 relaxation times of spheroids White arrows indicate spheroids.
  e. Image J will open a table describing the size of the selected area with respective mean, min and max values.
18. T1 Mapping.
  a. T1 Map has been created by inline manufacturer software “MapIt”.
  b. Open T1 Map DICOM file with Image J.
  c. To obtain a pseudo colored image select “LUT” (Look Up Table).
  d. Select LUT desired pseudo coloring (Figure 4).

**Figure 4:**
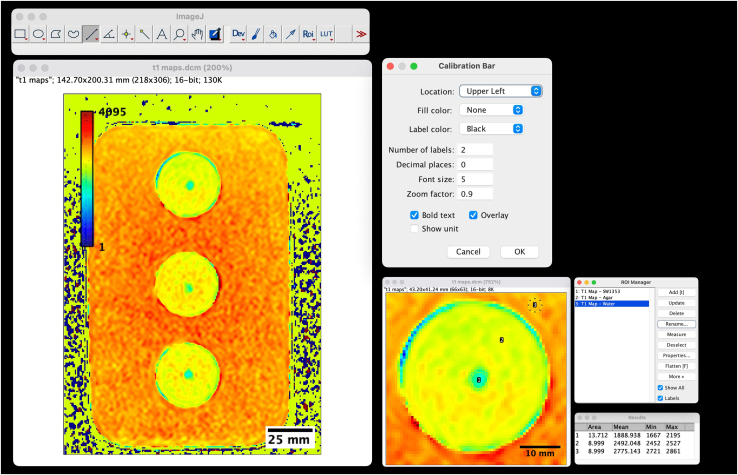
Color-encoded T1 map of SW1353 spheroid clusters The selected LUT is “JET”.
  e. Display color scheme by selecting Image > Color > Show LUT.
  f. Add calibration bar by selecting Analyze > Tools > Calibration Bar.
  g. Save Image.
19. T2 Mapping.
  a. T2 Map has been created by inline manufacturer software “MapIt”.
  b. Start Image J.
  c. Load T2 Map.
  d. Select the spheroids cluster with the oval or freehand selection.
  e. Open Analyze > Tool > ROI Manager to obtain relaxation times.
  f. For pseudo-coloring, see T1 Mapping.
20. Apparent Diffusion Coefficient.
  a. Start Image J.
  b. Load ADC-Image.
  c. Select the spheroids cluster with the oval or freehand selection.
  d. Open Analyze > Tool > ROI Manager.
21. MTR.
  a. Load your respective MTR images (“on” and “off”) in MR View&GO.
  b. Go to “MR calculations”.
  c. Subtract “on image” from “off image”.
  d. Multiply the subtraction image by 100, select “no scaling” in the configuration (Figure 5).

**Figure 5:**
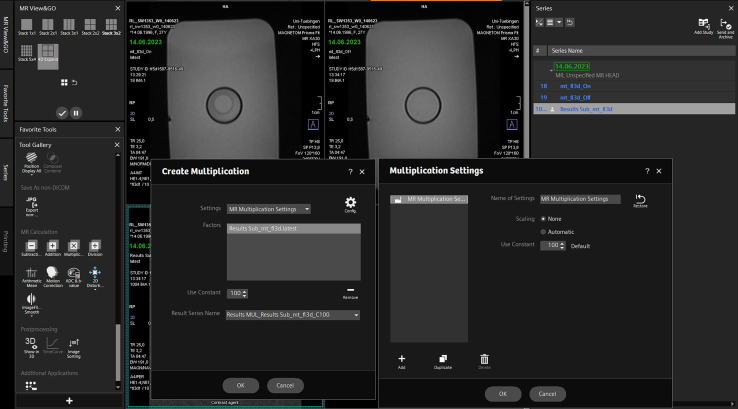
Creating an MTR image—Step 5: Multiplication of subtraction image
  e. Divide multiplication image (dividend) by “off image” (divisor).
  f. Save image.
  g. Start Image J.
  h. Load generated MTR image.
  i. Select the spheroids cluster with the oval or freehand selection.
  j. Open Analyze > Tool > ROI Manager.

## Expected outcomes

The protocol provides T1 and T2 relaxation times, as well as ADC and MTR values. As this new application is not yet widespread, reference values still need to be established for a large number of cell lines. However, the extant data suggest that the slight differences observed, resulting from factors such as cell size and vitality, are cell line specific.^1^

The analysis of all data enables the drawing of conclusions regarding cell viability:

ADC values decrease as cell death progresses, while the opposite effect is observed for MTR. The induction of cell death results in the release of the cytoplasm, leaving only the scaffold of the spheroid (comprising cell membranes and the extracellular matrix) intact. This results in a significant reduction in the apparent diffusion of water molecules and an increase in the measured proportion of macromolecules.^1^

The MRI scans can be repeated as often as required, thus enabling the observation of changes in the cells.

The findings of this study demonstrate that the utilization of a range of MRI sequences and features results in the acquisition of supplementary information pertaining to the composition of spheroids and the condition of the sample. This approach facilitates the attainment of results with greater expediency in comparison to conventional histological methods. This additional insight facilitates a non-destructive evaluation of emerging microenvironments and spheroid development, thereby enhancing our comprehension of their formation and progression. Moreover, it is possible to preserve valuable starting material, including stem cell spheroids, in the context of autologous chondrocyte transplantation. The present approach provides reliable insights and has the potential to serve as a valuable complement to current advanced techniques in monitoring cellular survival across diverse applications.

## Quantification and statistical analysis

**Figure 6:**
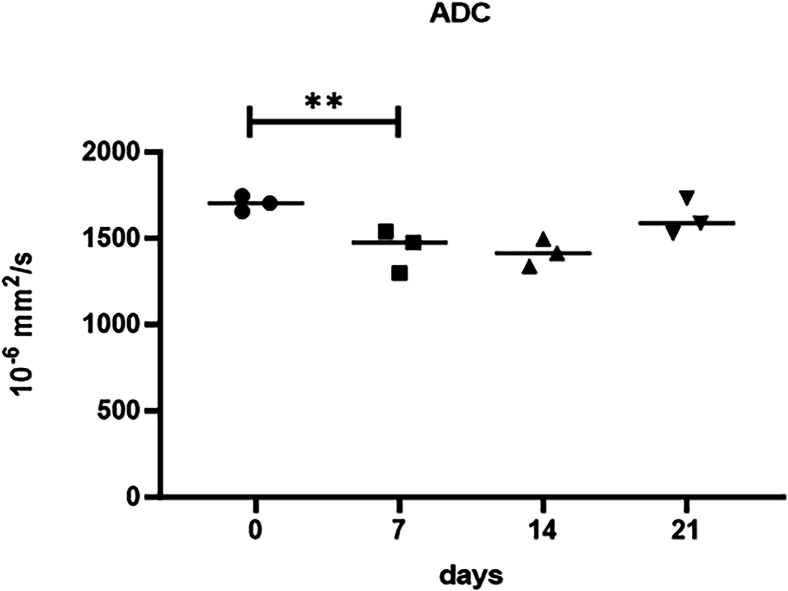
Temporal change of ADC values of SW1353 cell spheroids measured over a period of 21 days Data are represented as mean values and standard deviation from three independent experiments, ∗∗*p* < 0.01.

The acquired data (T1 and T2 relaxation times, ADC and MTR values) can be visualized through graphical representations, such as diagrams and plots, to enhance interpretability and facilitate comparative analysis (Figure 6).

For statistical evaluation, the consistency and reliability of replicates can be assessed by quantifying the standard deviation, providing a measure of variability within the dataset. Additionally, an unpaired t-test can be employed to identify statistically significant differences between measurement groups. GraphPad Prism serves as a robust tool for both graphical visualization and statistical analysis of the data.

**Figure 7:**
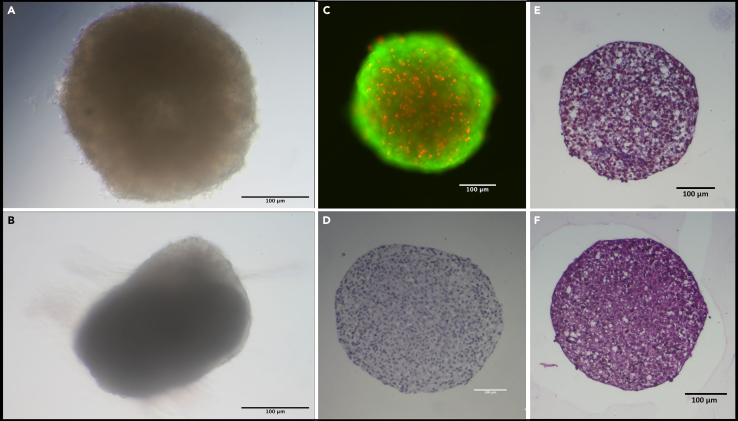
Microscopy images of SW1353 spheroids (A) Light microscopy of a newly formed spheroid. (B) Light microscopy of a 21-day old spheroid. (C) Merged Image of Calcein and Propidium iodide staining D: H&E Staining. (E) Elastika-van-Gieson Staining. (F) Masson-Trichrome Staining. Scale bars represent 100 μm. This figure is adapted from Wißmann et al.^1^

In order to extend the methodology to other cell lines, it is first necessary to compare the measurement results with those obtained using gold standard methods. Light microscopy (Figures 7A and 7B) facilitates the determination of cell integrity over time. Disintegration or fusion of the spheroids influences the data due to changes in the water content in the measuring area. For detailed compositions, the use of live-dead staining (Figure 7C) or histological staining (Figures 7D–7F) is recommended.

## Limitations

Cell spheroids can be cultivated within a specialized MR-compatible container and setup, enabling their visualization and non-invasive quantitative characterization using a clinical 3T MRI scanner. Nevertheless, the predictive power is limited by the lack of established reference values, and evaluating individual spheroids within a whole-body 3T MRI system is challenging due to limitations in spatial resolution. The respective voxel sizes of 0.5–1 mm^3^ cannot depict a single spheroid (100 μm). Achieving this level of detail would require specialized, higher-field MRI units, which are generally less accessible to clinical researchers.

The protocol was established and evaluated on a whole-body MRI system. It is noteworthy that analogous protocols and measurement parameters can be utilized on other whole-body MRI systems, including those from different manufacturers (although there may be variations in sequence nomenclature).

High-field MRI systems employed for animal examinations frequently facilitate enhanced spatial resolution. However, they also constrain the available space for samples. Consequently, when conducting measurements on cell spheroids with small animal MRI systems, it is essential to adapt the dimensions of the experimental setup accordingly.

It should also be noted that the resources required for implementing the proposed MRI protocol are considerably higher than those needed for conventional techniques. MRI-based approaches typically demand more sophisticated equipment, longer acquisition times, and greater computational resources for image reconstruction and analysis compared to standard live-dead staining and fluorescence microscopy. These factors can limit throughput and accessibility, especially for large-scale or time-sensitive studies. Furthermore, alternative techniques such as live-dead staining of spheroids (e.g., using calcein-AM and Helix NP Blue) offer cost-effective, less resource-intensive, and relatively non-toxic means of assessing spheroid viability and therapy response. These methods can be applied for longitudinal monitoring without additional handling or significant perturbation of the spheroids, and are compatible with standard widefield or confocal microscopy systems.^19^

## Troubleshooting

### Problem 1

Cells do not form spheroids.

### Potential solution

Spheroid formation depends on cell line. Check if cell line generally forms spheroids. If yes, extend the cultivation in ultra-low attachment plates.

### Problem 2

Quantitative MR features differ in replicative measurements.

### Potential solution

Temperature might not be consistent, check the temperature of ddH_2_O and avoid long storage times outside the incubator.

## Resource availability

### Lead contact

Further information and requests for resources and reagents should be directed to and will be fulfilled by the lead contact, Rebecca Wißmann (rebecca.wissmann@med.uni-tuebingen.de).

### Technical contact

For detailed technical questions, please reach out to the technical contact, Dr. Petros Martirosian (petros.martirosian@med.uni-tuebingen.de).

### Materials availability

All cell lines and reagents are commercially available.

### Data and code availability

This paper does not report original code. All utilized programs are either freeware or commercially available.

## Acknowledgments

We would like to express our gratitude to Rosa Riester for her invaluable contributions to our project, drawing upon her considerable expertise and experience. We would like to express our gratitude to Prof. Dr. Konstantin Nikolaou and the Department of Diagnostic and Interventional Radiology for their financial support of this research project. We acknowledge support from the Open Access Publishing Fund of the University of Tübingen.

## Author contributions

Conceptualization, R.W., S.E., and F.S.; methodology, R.W., P.M., M.D., and S.E.; investigation, R.W. and P.M.; writing – original draft, R.W., P.M., and F.S.; writing – review and editing, M.D., S.E., and F.S.; resources, S.E. and F.S.; supervision, M.D., S.E., P.M., and F.S.

## Declaration of interests

The authors declare no competing interests.

## Declaration of generative AI and AI-assisted technologies in the writing process

During the preparation of this work, the authors used DeepL in order to enhance the readability of the manuscript and eliminate language issues. After using this service, the authors reviewed and edited the content as needed and take full responsibility for the content of the publication.
